# Surgical Outcomes of a Second Ahmed Glaucoma Valve Implantation in Asian Eyes with Refractory Glaucoma

**DOI:** 10.1155/2020/8741301

**Published:** 2020-03-23

**Authors:** Sze Chuan Ong, Maria Cecilia Aquino, Paul Chew, Victor Koh

**Affiliations:** ^1^Yong Loo Lin School of Medicine, National University of Singapore, 117597, Singapore; ^2^Department of Ophthalmology, National University Hospital, 119228, Singapore

## Abstract

**Results:**

The cumulative failure rates were 9.5%, 20.0%, 32.5%, and 46.0% at six months, one year, two years, and three years of follow-up. At final follow-up, complete success and qualified success rates were 23.8% and 33.3%, respectively; mean IOP and number of medications decreased by 5.6 mmHg (23.9%) and 1.7 mmHg (54.8%), respectively, from preoperative baseline (*P* < 0.01). More common postoperative complications included hypertensive phase (38.1%), corneal decompensation (23.8%), and tube exposure (14.3%).

**Conclusion:**

An additional AGV implant had good short and modest long-term effectiveness in reducing IOP following a failed glaucoma tube shunt in Asian eyes, with the mentioned common postoperative complications to be actively monitored and managed.

## 1. Introduction

Glaucoma tube shunts are effective in lowering intraocular pressure (IOP) for eyes with refractory glaucoma [1–4]. One of the most common implant is Ahmed glaucoma valve (AGV; New World Medical, Inc., CA), a glaucoma drainage device (GDD) that drains aqueous humour into a 184 mm^2^ plate close to the equator of the eye. The tube versus trabeculectomy (TVT) study showed that the glaucoma tube shunt group experienced fewer early postoperative complications, were less likely to undergo additional glaucoma surgery, and had higher success rates when compared to using trabeculectomy with MMC [5, 6]. This has consequently encouraged the utility of GDD for both primary and secondary glaucoma [7, 8].

If the first glaucoma tube shunt implant fails to control IOP adequately, there are limited surgical options for subsequent IOP control which includes revision of the primary tube shunt, implantation of a second GDD, and cyclodestructive laser procedures. Shah et al. reported that additional tube shunt has better IOP control than tube shunt revision by excision of an encapsulated bleb [9]. Levinson et al. demonstrated that while both second GDDs and cyclodestructive procedures effectively reduce IOP after a failed primary GDD, a second GDD had better long-term success rates [10, 11]. The growing use of GDDs and increased lifespan of our glaucoma patients has also contributed to the use of sequential GDD to lower IOP and reduce glaucomatous optic neuropathy [12, 13].

Currently, several studies have reported the surgical outcomes of a second AGV, with varying success, but there is a lack of studies in an Asian glaucoma population. The purpose of this study is thus to evaluate the short- and long-term outcomes of a second AGV implant and to define risk factors for failure of the second AGV implant in an Asian population.

## 2. Materials and Methods

This is a retrospective study that is approved by the National Health Group Domain Specific Review Board. It was conducted in accordance with the ethical principles that have their origin in the Declaration of Helsinki and that are consistent with the Singapore Good Clinical Practice and the applicable regulatory requirements.

We reviewed the medical records of 569 patients who consecutively underwent an AGV implantation between January 2008 and March 2019, at the Eye Surgery Centre, National University Hospital, Singapore. In total, 32 eyes of 31 patients received a second AGV implantation (FP7 model) after their first GDD has failed to control the IOP. Patients who underwent a second AGV implantation during this period were excluded from this study if their postoperative follow-up period was less than 3 months, or if their medical records were missing or insufficient for the purposes of this study.

The demographic and ocular characteristic data collected included the age, gender, race, eye laterality, history of hypertension and diabetes mellitus, glaucoma diagnosis, history of trabeculectomy, date of first GDD implantation, lens status (phakic, pseudophakic, or aphakic), visual field results, clinical vertical cup-to-disc ratio, best corrected visual acuity (BCVA), IOP, and the number of IOP-lowering medications. Intraoperative complications and placement location of the second AGV were also collected. Postoperative data collected included BCVA, IOP, and IOP-lowering medications at each follow-up visit and any postoperative complications and surgical interventions.

### 2.1. Surgical Technique and Postoperative Care

After informed consent, all the patients underwent AGV implantation at a selected quadrant which did not have significant conjunctiva scarring or preexisting scleral thinning. The AGV implants were all primed with balanced salt solution injected from a blunt cannula. After adequate conjunctival peritomy and dissection, the anterior portion of the AGV plate was sutured to the sclera (avoiding the recti muscles) between 8.5 and 9.0 mm posterior to the limbus. After anterior chamber paracentesis was performed, a 25 or 23G needle was used to create a scleral track into the anterior chamber. The tube was shortened and inserted into the anterior chamber just above the iris plane or into the mid-anterior chamber depth. The tube was sutured down onto the sclera and a piece of sterilised bovine pericardium (Tutopatch; RTI Surgical Holdings, Alachua, Florida) was fixated over the tube. The conjunctiva was approximated and sutured to the limbus.

### 2.2. Outcome Measures

The primary outcome measure was the surgical success rate, classified as complete success (IOP ≥5 mmHg and ≤21 mmHg without the use of IOP-lowering medications) and qualified success (IOP ≥5 mmHg and ≤21 mmHg with the use of IOP-lowering medications). Failure was defined as any of the following: IOP >21 mmHg or <5 mmHg for 2 or more consecutive visits after three months of follow-up, surgical intervention for IOP-related indications, removal of the second AGV implant, or loss of light perception.

We defined early postoperative complications as those occurring within the first 12 postoperative weeks, and late postoperative complications as those occurring after this time period. We defined the presence of a hypertensive phase as having an IOP >21 mmHg in the first three postoperative months [14, 15].

### 2.3. Statistical Methods

Kaplan–Meier survival analysis was applied to evaluate the cumulative failure rates based on the criteria above. All BCVA measured by the Snellen chart were converted to logMAR (logarithm of the minimum angle of resolution) for analysis, with any visual acuity worse than or equal to 6/60 on the Snellen chart being allocated a logMAR value of 1.00. Wilcoxon signed rank test was used to compare the continuous variables of IOP, number of medications, and BCVA at each follow-up time point with baseline levels.

Univariate and multivariate cox proportional-hazards regression analyses were used to evaluate risk factors for failure. The following preoperative characteristics were evaluated as possible risk factors: age, gender, race, history of hypertension and diabetes mellitus, glaucoma diagnosis (primary vs. secondary glaucoma), history of trabeculectomy surgery, duration between the first and second GDD implantation, lens status, visual field results, clinical vertical cup-to-disc ratio, best corrected visual acuity (BCVA), IOP, and number of IOP controlling medications; other risk factors considered were intraoperative location of second AGV implantation and the presence of postoperative corneal graft. Risk factors with a *P* value of <0.2 in univariate analysis were included in the multivariate analysis. Risk factors with a *P* value of <0.05 in the multivariate analysis were considered statistically significant.

## 3. Results

### 3.1. Patient Demographics and Characteristics

The records of 32 eyes of 31 patients were reviewed. 11 eyes were excluded (four eyes had a follow-up duration of less than three months; seven eyes had insufficient medical records and data for the purposes of this study). In total, 21 eyes of 20 patients with a second AGV implantation were included in the final analysis of this study. Demographic characteristics of the study sample are shown in Table 1. The mean (±SD) age of the patients at the time of second AGV implantation was 48.8 ± 17.6 years (range: 12–83 years). The mean (±SD) and median duration of follow-up was 4.1 ± 2.6 years and 3.6 years (range: 0.7–9.2 years). Of the 21 eyes, up to one-quarter of the eyes had both previous failed trabeculectomy and GDD surgeries and half of them had secondary glaucoma.

**Figure 1:**
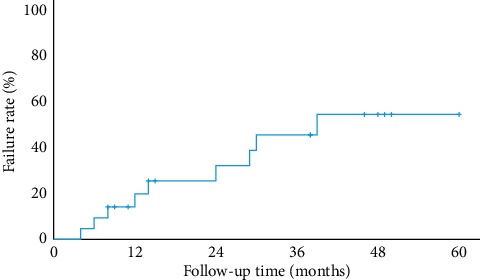
Kaplan–Meier survival curve at six months and one, two, and three years after surgery.

The Kaplan–Meier survival analysis demonstrated cumulative failure rates of (standard errors) of 9.5% (6.4%), 20.0% (9.0%), 32.5% (11.2%), and 46.0% (12.4%) at six months, one year, two years, and three years of follow-up according to the criteria defined above (Figure 1). The median time of survival was 3.3 years.

Table 2 shows the success rates at various follow-up time points. In all, seven eyes (77.8%) failed as they had IOPs of >21 mmHg on two consecutive visits after three months, and two eyes (22.2%) failed for undergoing a reintervention for IOP-related indications (one underwent tube flushing; the other one underwent micropulse transscleral cyclophototherapy). No eyes experienced hypotony of <5 mmHg on two consecutive visits after three months, loss of light perception, or removal of their second AGV implant.

### 3.2. Intraocular Pressure and Number of Medications

**Figure 2:**
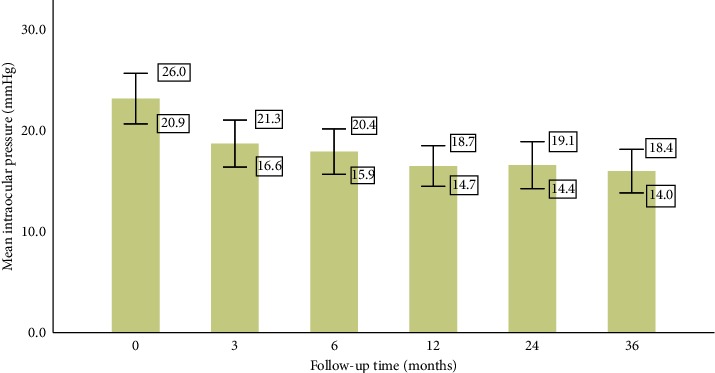
Mean intraocular pressure at preoperative baseline and at various follow-up time points after the implantation of the second Ahmed glaucoma valve. Whiskers represent the 95% confidence interval range of values.

**Figure 3:**
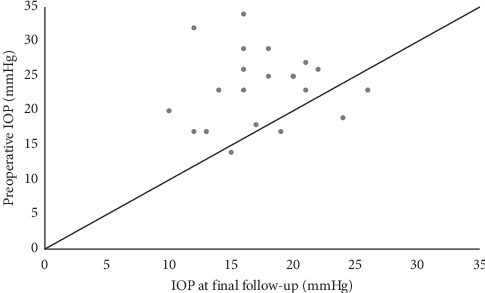
Scatter plot comparing preoperative intraocular pressure (IOP) with IOP at final follow-up. Each dotted symbol represents a single subject. Dots that are above the diagonal line represent subjects with lower IOP at final follow-up compared to their preoperative baseline IOP.

The mean (±SD) IOP decreased from a baseline preoperative value of 23.4 ± 5.4 mmHg to 19.0 ± 5.0 mmHg (*P*=0.006) at three months, 18.1 ± 5.0 mmHg (*P*=0.003) at six months, 16.7 ± 3.9 mmHg (*P*=0.003) at one year, 16.8 ± 4.1 mmHg (*P*=0.003) at two years, and 16.2 ± 3.0 mmHg (*P*=0.011) at three years (Figure 2). The mean IOP at the point of last follow-up was 17.8 ± 5.0 mmHg, and the mean reduction of 5.6 mmHg (23.9%) from baseline IOP levels was statistically significant (*P* < 0.001). Out of 21 subjects, 17 (81.0%) have a lower IOP at final follow-up than their baseline preoperative IOP (Figure 3).

**Figure 4:**
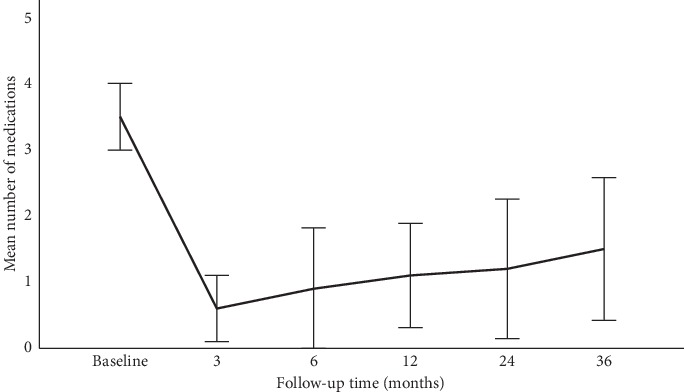
Mean number of medications at preoperative baseline and at subsequent postoperative time points after the implantation of the second Ahmed glaucoma valve. Whiskers represent the 95% confidence interval range of values.

The mean (±SD) number of medications decreased from a baseline preoperative value of 3.1 ± 0.8 to 0.6 ± 0.9 (*P* < 0.001) at three months, 0.7 ± 1.1 (*P* < 0.001) at six months, 0.8 ± 1.0 (*P*=0.001) at one year, 1.0 ± 1.4 (*P*=0.003) at two years, and 1.5 ± 1.5 (*P*=0.023) at three years (Figure 4). The mean number of medications at the point of last follow-up was 1.4 ± 1.5, and the reduction of 1.7 in mean number of medications (54.8%) was a statistically significant decrease from baseline (*P* < 0.001).

### 3.3. Best Corrected Visual Acuity

The mean (±SD) BCVA increased from a baseline value of 0.47 ± 0.34 logMAR to 0.53 ± 0.29 logMAR at three months, 0.56 ± 0.35 logMAR at six months, 0.49 ± 0.37 logMAR at one year, 0.60 ± 0.37 logMAR at two years, and 0.62 ± 0.40 logMAR at three years of follow-up. The increase in BCVA at each time point compared to baseline was statistically insignificant (*P* > 0.05, Wilcoxon signed rank test).

### 3.4. Complications and Reinterventions

There were no intraoperative complications. Postoperatively, eight eyes (38.1%) experienced a hypertensive phase after the second AGV implantation. There were three eyes (14.3%) with tube exposure and were repaired with a corneal graft patch surgery. In total, two eyes (9.5%) developed corneal decompensation and bullous keratopathy, both of which subsequently underwent corneal endothelial transplant surgery. In total, nine eyes (42.9%) required further IOP-lowering interventions (eight eyes underwent micropulse transscleral cyclophototherapy, two eyes underwent tube flushing, and three eyes received a subsequent third GDD implantation). There were no cases of diplopia or ocular movement limitation and no AGVs were explanted.

### 3.5. Risk Factors for Failure of Second AGV

The risk factors identified with univariate analysis (*P* < 0.2) were a history of diabetes mellitus (*P*=0.19), reduced cup-to-disc ratio (*P*=0.01) and visual field index (*P*=0.17), higher preoperative IOP (*P*=0.14), and higher number of preoperative medications (*P*=0.19). However, there were no statistically significant risk factors for failure identified using the multivariate Cox proportional hazards model (*P* < 0.05).

## 4. Discussion

In eyes with refractory glaucoma, our results showed that the implantation of an additional FP7 AGV following a previous failed GDD is relatively effective in controlling IOP for Asian eyes with glaucoma but there were considerable complications and additional interventions required for IOP control. Preoperatively, our patients had a mean IOP of 23.4 ± 5.4 mmHg and were on a mean number of 3.1 ± 0.6 IOP-lowering medications. At the last follow-up after the second AGV implantation, patients had a mean IOP of 17.8 ± 5.0 mmHg, which was a statistically significant decrease from baseline by 5.6 mmHg (23.9%), while being on fewer IOP-lowering medications. Patients had a statistically significant reduction in both their IOP and number of medications at six months, one year, two years, and three years of follow-up.

The cumulative failure rates of our study determined by Kaplan–Meier analysis was 9.5%, 20.0%, 32.5%, and 46.0% at six months, one year, two years, and three years of follow-up, respectively. Whilst the short-term success rates were good, the longer-term success rates of a second AGV implantation were relatively modest. We reviewed studies which investigated the surgical outcomes of second GDDs, specifically AGV (Table 3). These studies were not carried out in Asian countries, with the exception of one, Ko et al. in Korea. They reported similar success rates ranging from 62.9% to 87.0% at one year, 56.6% to 80.0% at two years, and 52.0% to 57.0% at three years of follow-up [16–20]. The study on Asian eyes by Ko et al. showed very similar success rates to our results. Our study showed higher success rates at all three intervals compared to that of Jiménez-Román et al., possibly due to the high proportion of S2 polypropylene models as the second AGV in their study; polypropylene models have been reported to have poorer survival estimates than the silicone models [19, 21].

The success rates of the second AGV at the last follow-up in these studies ranged from 65.2% (Ko et al.) to 84.2% (Smith et al.), while our study had a lower success rate of 57.1% at the last follow-up [16–18]. This could be attributed to a longer mean follow-up time of 49.2 months in our study as compared to the studies evaluated (range: 21.4 months–37.8 months), which led to a greater proportion of AGV failure over time. The higher success rate of 84.2% reported by Smith et al. may also be due to a higher proportion of primary open-angle glaucoma (47.4%) in their study compared to ours (33.3%), which might be less refractory to glaucoma treatment compared to secondary glaucoma [17]. The proportion of successful AGVs which still required medications in our study (58.3%) was similar to that reported by Nilforushan et al. (51.2%). This suggests that patients still require IOP-lowering medications after a second AGV.

The mean IOP reduction for our study (23.9%) was lower than that reported by the other studies, which ranged from 42.0% to 52.9%. This could be due to differences in study methodology, population demographics, and types of glaucoma drainage implants. Preoperative IOP in our study was measured while patients were on medication; the other studies could be measuring preoperative unmedicated IOP, thus leading to a higher preoperative IOP value and a greater degree of IOP reduction postoperatively. In their studies, Ko et al. and Smith et al. included second AGV implants which were of FP8 and S2 models; however, this may not fully account for the difference in IOP reduction. Koh et al. have shown that there is no difference in IOP reduction between FP7 and FP8 models [22]. Mackenzie et al. have also reported that silicon-based (FP7) models are instead superior in reducing IOP when compared to propylene based (S2) models [12, 13].

Previous studies demonstrating the success rates of initial AGVs yielded similar results. Their cumulative success rates ranged from 75.0% to 87.0% at one year, 60.6% to 75.0% at two years, and 54.0% to 66.0% at three years [2, 23–27]. Despite being a second AGV implantation, our success rates are comparable to that of initial AGVs in current literature. This could reflect the long-term efficacy of a second FP7 AGV as a treatment option following a failed first GDD, as supported by Levinson et al. [10, 11]. Several other studies have also evaluated the outcomes of second GDDs in general (not confined to AGV implants). Burgoyne et al. have demonstrated a success rate of 50% at the last follow-up, with a mean IOP reduction of 33.0% from baseline levels [28]. Hu et al. have also reported a mean IOP reduction of 32.6% following a second GDD implantation [29]. Compared to our study, they demonstrated higher mean IOP reduction following a second GDD implantation. It should be noted that a substantial proportion of the second GDD used in their studies were nonvalved implants (Baerveldt or Molteno implants), which has been shown to result in a greater IOP reduction [30].

In our study, there were no serious intraoperative or postoperative complications which led to a loss of light perception. However, the most frequent early postoperative event was the development of a hypertensive phase, which occurred in 38.1% (*n* = 8) of the eyes. This was similarly reported by Jiménez-Román et al. but at a lower rate of 10.3% [20]. The hypertensive phase might be attributed to bleb encapsulation mediated by inflammatory cytokines such as interleukin-6, interleukin-10, and chemokine C-X-C motif ligand 1 [15], which could be even higher in the presence of a previous first GDD tube. Nouri-Mahdavi and Caprioli have demonstrated that while hypertensive phase occurs frequently after an AGV implantation, it resolves only in a minority of eyes [31]. There is thus impetus for closer monitoring and quicker resolution of hypertensive phases, especially among the Asian population.

We found tube exposure of the second AGV to be the most frequent late postoperative complication, occurring in three eyes (14.3%). A widely proposed mechanism of tube erosion and exposure is a combination of mechanical forces of the eyelid and tension of the tube on the conjunctiva [32]. The high incidence of tube exposure in a sequential tube could be attributed to a greater degree of conjunctiva breakdown from the surgical trauma and conjunctival stretching over the second implant. Byun et al. demonstrated that eyes with one or more prior ocular surgeries had a significantly higher risk of AGV exposure (odds ratio, 9.06; *P*=0.006) [33]. Levinson et al. have also reported that the rate of tube exposure in sequential GDDs and primary GDDs were 13.1% and 5.8%, respectively, although this increased risk did not approach statistical significance [34]. When comparing with other Western studies evaluating second AGVs, Fatehi et al. and Jiménez-Román et al. reported tube exposure rates of 2.7% and 1.7%, respectively while Nilforushan et al. and Smith et al. did not report any incidence of tube exposure [17–20]. The higher rate of tube exposure in our study could be secondary to the tighter eyelids found in Asian eyes, which contribute to pressure necrosis of the conjunctiva over the implant. Choo et al. in their study on the outcomes of first AGV in Asian eyes have also demonstrated that tube exposure rates are much higher when compared to first AGV in Western eyes [35].

Another significant late postoperative complication which we observed was corneal decompensation and bullous keratopathy (9.5%). Jiménez-Román et al., Ko et al., and Smith et al., whose studies included S2 AGV models as well, reported similar findings, albeit at varying frequencies of 17.2%, 13.0%, and 5.3%, respectively [16, 17, 20]. While studies comparing silicone (FP7) and polypropylene (S2) models have showed higher general complication rates in the latter, there were no statistically significant differences reported specifically for the risk of developing corneal edema [12, 21, 36]. Shah et al. also reported higher frequencies of corneal edema after a second GDD implantation, at 45.0% and 43.0%, respectively [9, 28]. However, these two studies included other GDD types apart from the AGV, most notably the Baerdvelt tube which has a higher rate of complications [30]. Nevertheless, corneal decompensation is a frequent complication and should therefore be taken into account when deciding on an additional AGV of any model.

Ko et al. reported prior trabeculectomy to be a significant risk factor for failure of the second AGV (relative risk, 1.78; *P*=0.027) [16]. Fatehi et al. demonstrated that a lower number of preoperative medications (hazard ratio, 0.57; *P* < 0.001) and a younger age at surgery (hazard ratio, 0.82; *P*=0.029) were risk factors for failure, respectively [19, 20]. Meanwhile, our study analysis showed no statistically significant risk factors for failure in Asian eyes despite evaluating the patient characteristics and data which we collected. This could be due to our small sample size, and a larger study is needed to confirm the results of our study.

There were some limitations of our study. The small sample size could have affected the accuracy of the Cox proportional hazard model analysis, which could explain the lack of risk factors for failure identified. Our study sample consisted mostly of male eyes without prior trabeculectomy, which would limit its generalizability. The retrospective nature of the study design was also a limitation in evaluating outcomes, success rates, and determining causality of risk factors. A larger, prospective randomised study over a long follow-up period is needed to confirm the results of this study.

## 5. Conclusion

In conclusion, the implantation of a second AGV following a previous failed AGV implantation has good short-term but modest long-term success rates in reducing IOP. Most of these patients will still require IOP-lowering medications, but with a fewer number than preoperative baseline, for optimal IOP control after the second AGV surgery. Hypertensive phase, tube exposure, and corneal decompensation are frequent complications which should be actively monitored and managed, especially among the Asian population.

## Figures and Tables

**Table 1:** Demographic characteristics of patients and eyes in the study sample at the time of the second Ahmed glaucoma valve implantation.

Characteristic	Results
Number of eyes (patient)	21 (20)
Age, mean years (SD^†^)	48.8 (17.6)
Gender, *n* (%) patients	
Male	18 (90.0)
Female	2 (10.0)
Race, *n* (%) patients	
Chinese	12 (60.0)
Malay	2 (10.0)
Indian	3 (15.0)
Others	3 (15.0)
Prior trabeculectomy done, *n* (%) eyes	
Yes	5 (23.8)
No	16 (76.2)
Diagnosis, *n* (%) eyes	
Primary open-angle glaucoma (POAG)	7 (33.3)
Neovascular glaucoma	1 (4.8)
Traumatic glaucoma	2 (9.5)
Uveitic glaucoma	4 (19.0)
Aphakic glaucoma	1 (4.8)
Silicone oil induced glaucoma	2 (9.5)
Congenital glaucoma	2 (9.5)
Juvenile glaucoma	1 (4.8)
Glaucoma secondary to Rieger's anomaly	1 (4.8)
Lens status, *n* (%) eyes	
Phakic	3 (14.2)
Pseudophakic	17 (81.0)
Aphakic	1 (4.8)
First GDD^‡^ model, *n* (%) eyes	
AGV^§^—FP7	16 (76.2)
AGV—S2	3 (14.4)
Baerveldt shunt	1 (4.8)
Unknown	1 (4.8)
Time between first GDD and second AGV implantation, mean years (SD)	5.4 (0.7)
Second AGV location, *n* (%) eyes	
Superotemporal	5 (23.7)
Superonasal	9 (42.9)
Inferotemporal	6 (28.6)
Inferonasal	1 (4.8)

^†^SD = standard deviation; ^‡^GDD = glaucoma drainage device, ^§^AGV = Ahmed glaucoma valve.

**Table 2:** Success rates of second Ahmed glaucoma valve at postoperative follow-up time points.

	One year (*n* = 18)	Two years (*n* = 16)	Three years (*n* = 15)	Final follow-up (*n* = 21)
Complete success, *n* (%)	8 (44.5)	6 (37.5)	4 (26.7)	5 (23.8)
Qualified success, *n* (%)	1 (12.5)	1 (6.2)	3 (20.0)	7 (33.3)
Failure, *n* (%)	9 (50.0)	9 (56.3)	8 (53.3)	9 (42.9)

**Table 3:** Comparison of studies evaluating success rates and surgical outcomes of second Ahmed glaucoma valve surgeries.

Authors	Ko et al. [16]	Smith et al. [17]	Nilforushan et al. [18]	Fatehi et al. [19]	Jiménez-Román et al. [20]	Our study
Number of eyes	23	19	36	110	58	21
Mean age (years)	44.5	58.0	32.7	63.8	46.1	48.8
Gender, male (%)	56.6	47.4	44.4	49.0	38.0	90.0
Mean follow-up (months)	37.8	38.8	21.4	59.6	18.4	49.2
First GDD type	17 FP7 (73.9%) 6 FP8 (26.1%)	19 FP7/S2 (unknown proportion)	—	—	6 S3 (10.3%) 50 S2 (86.2%) 2 FP7 (3.5%)	16 FP7 (76.2%) 3 S2 (14.4%) 1 Baerveldt (4.8%) 1 unknown (4.8%)
Second GDD type (%)	2 FP7 (8.7%) 21 FP8 (91.3%)	19 FP7/S2 (unknown proportion)	36 FP7 (100%)	55 FP7 (50%) 55 S2 (50%)	50 S2 (86.2%) 2 FP7 (3.5%)	21 FP7 (100%)
Mean preoperative IOP (mmHg)	39.3	18.8	26.9	25.7	27.6	23.4
Mean IOP reduction (mmHg)^†^	20.8 (52.9%)	7.9 (42.0%)	13.6 (50.6%)	11.3 (44.1%)	12.7 (45.0%)	5.6 (23.9%)
Success rates^‡^	87.0% (1 year) 70.0% (2 years) 52.0% (3 years)	—	94.0% (6 months) 85.0% (1 year) 80.0% (18 months) 53.0% (42 months)	70% (1 year) 63% (3 years) 57% (5 years)	62.9% (12 months) 56.6% (30 months)	80.0% (1 year) 67.5% (2 years) 54.0% (3 years)
Success^†^	15 (65.2%)	16 (84.2%)	27 (75.0%)	—	—	12 (57.1%)
Failure^†^	8 (34.8%)	3 (15.8%)	9 (25.0%)	—	—	9 (42.9%)

^†^Calculated at the last follow-up, ^‡^criterion for success: IOP ≥5 mmHg and ≤21 mmHg, with ≥20% reduction in IOP.

## Data Availability

The data used to support the findings of this study are restricted by the Domain Specific Review Board in order to protect patient privacy. Data are available from the corresponding author for researchers who meet the criteria for access to confidential data.

## References

[1] Ayyala R. S., Zurakowski D., Smith J. A. (1998). A clinical study of the Ahmed glaucoma valve implant in advanced glaucoma. *Ophthalmology*.

[2] Lee C. K., Ma K. T., Hong Y. J., Kim C. Y. (2017). Long-term clinical outcomes of Ahmed valve implantation in patients with refractory glaucoma. *PLoS One*.

[3] Englert J. A., Freedman S. F., Cox T. A. (1999). The Ahmed valve in refractory pediatric glaucoma. *American Journal of Ophthalmology*.

[4] Papadaki T. G., Zacharopoulos I. P., Pasquale L. R., Christen W. B., Netland P. A., Foster C. S. (2007). Long-term results of Ahmed glaucoma valve implantation for uveitic glaucoma. *American Journal of Ophthalmology*.

[5] Gedde S. J., Schiffman J. C., Feuer W. J., Herndon L. W., Brandt J. D., Budenz D. L. (2012). Treatment outcomes in the tube versus trabeculectomy (TVT) study after five years of follow-up. *American Journal of Ophthalmology*.

[6] Gedde S. J., Herndon L. W., Brandt J. D., Budenz D. L., Feuer W. J., Schiffman J. C. (2012). Postoperative complications in the tube versus trabeculectomy (TVT) study during five years of follow-up. *American Journal of Ophthalmology*.

[7] Gedde S. J., Singh K., Schiffman J. C., Feuer W. J. (2012). The tube versus trabeculectomy study: interpretation of results and application to clinical practice. *Current Opinion in Ophthalmology*.

[8] Vinod K., Gedde S. J., Feuer W. J. (2017). Practice preferences for glaucoma surgery: a survey of the American Glaucoma Society. *Journal of Glaucoma*.

[9] Shah A. A., WuDunn D., Cantor L. B. (2000). Shunt revision versus additional tube shunt implantation after failed tube shunt surgery in refractory glaucoma. *American Journal of Ophthalmology*.

[10] Levinson J. D., Giangiacomo A. L., Beck A. D. (2017). A comparison of sequential glaucoma drainage device implantation versus cyclophotocoagulation following failure of a primary drainage device. *Journal of Glaucoma*.

[11] Wang M. Y., Patel K., Blieden L. S. (2017). Comparison of efficacy and complications of cyclophotocoagulation and second glaucoma drainage device after initial glaucoma drainage device failure. *Journal of Glaucoma*.

[12] Mackenzie P. J., Schertzer R. M., Isbister C. M. (2007). Comparison of silicone and polypropylene Ahmed glaucoma valves: two-year follow-up. *Canadian Journal of Ophthalmology*.

[13] Hinkle D. M., Zurakowski D., Ayyala R. S. (2007). A comparison of the polypropylene plate Ahmed glaucoma valve to the silicone plate Ahmed glaucoma flexible valve. *European Journal of Ophthalmology*.

[14] Jung K. I., Park H., Jung Y., Park C. K. (2015). Serial changes in the bleb wall after glaucoma drainage implant surgery: characteristics during the hypertensive phase. *Acta Ophthalmologica*.

[15] Fargione R. A., Tansuebchueasai N., Lee R., Tai T. Y. T. (2019). Etiology and management of the hypertensive phase in glaucoma drainage-device surgery. *Survey of Ophthalmology*.

[16] Ko S. J., Hwang Y. H., Ahn S. I., Kim H. K. (2016). Surgical outcomes of additional ahmed glaucoma valve implantation in refractory glaucoma. *Journal of Glaucoma*.

[17] Smith M., Buys Y. M., Trope G. E. (2009). Second Ahmed valve insertion in the same eye. *Journal of Glaucoma*.

[18] Yadgari M., Nilforushan N., Jazayeri A. A., Karimi N. (2016). Evaluation of success after second Ahmed glaucoma valve implantation. *Indian Journal of Ophthalmology*.

[19] Fatehi N., Morales E., Parivisutt N., Alizadeh R., Ang G., Caprioli J. (2018). Long-term outcome of second ahmed valves in adult glaucoma. *American Journal of Ophthalmology*.

[20] Jiménez-Román J., Gil-Carrasco F., Costa V. P. (2016). Intraocular pressure control after the implantation of a second Ahmed glaucoma valve. *International Ophthalmology*.

[21] Ishida K., Netland P. A., Costa V. P., Shiroma L., Khan B., Ahmed I. K. (2006). Comparison of polypropylene and silicone ahmed glaucoma valves. *Ophthalmology*.

[22] Koh K. M., Hwang Y. H., Jung J. J., Sohn Y. H., Kim H. K. (2013). Comparison of the outcome of silicone Ahmed glaucoma valve implantation with a surface area between 96 and 184 mm^2^ in adult eyes. *Korean Journal of Ophthalmology*.

[23] Coleman A. L., Smyth R. J., Wilson M. R., Tam M. (1997). Initial clinical experience with the Ahmed glaucoma valve implant in pediatric patients. *Archives of Ophthalmology*.

[24] Topouzis F., Coleman A. L., Choplin N. (1999). Follow-up of the original cohort with the Ahmed glaucoma valve implant. *American Journal of Ophthalmology*.

[25] Schimiti R. B., Abe R. Y., Tavares C. M., Vasconcellos J. P., Costa V. P. (2016). Intraocular pressure control after implantation of an ahmed glaucoma valve in eyes with a failed trabeculectomy. *Journal of Current Glaucoma Practice*.

[26] Huang M. C., Netland P. A., Coleman A. L., Siegner S. W., Moster M. R., Hill R. A. (1999). Intermediate-term clinical experience with the ahmed glaucoma valve implant. *American Journal of Ophthalmology*.

[27] Souza C., Tran D. H., Loman J., Law S. K., Coleman A. L., Caprioli J. (2007). Long-term outcomes of Ahmed glaucoma valve implantation in refractory glaucomas. *American Journal of Ophthalmology*.

[28] Burgoyne J. K., WuDunn D., Lakhani V., Cantor L. B. (2000). Outcomes of sequential tube shunts in complicated glaucoma. *Ophthalmology*.

[29] Hu W. D., Moster M. R., Zheng C. X. (2016). Outcomes of sequential glaucoma drainage implants in refractory glaucoma. *Journal of Glaucoma*.

[30] Christakis P. G., Zhang D., Budenz D. L., Barton K., Tsai J. C., Ahmed I. I. K. (2017). Five-year pooled data analysis of the Ahmed Baerveldt comparison study and the Ahmed versus Baerveldt study. *American Journal of Ophthalmology*.

[31] Nouri-Mahdavi K., Caprioli J. (2003). Evaluation of the hypertensive phase after insertion of the ahmed glaucoma valve. *American Journal of Ophthalmology*.

[32] Heuer D. K., Budenz D., Coleman A. (2001). Aqueous shunt tube erosion. *Journal of Glaucoma*.

[33] Byun Y. S., Lee N. Y., Park C. K. (2009). Risk factors of implant exposure outside the conjunctiva after Ahmed glaucoma valve implantation. *Japanese Journal of Ophthalmology*.

[34] Levinson J. D., Giangiacomo A. L., Beck A. D. (2015). Glaucoma drainage devices: risk of exposure and infection. *American Journal of Ophthalmology*.

[35] Choo J. Q. H., Chen Z. D., Koh V. (2018). Outcomes and complications of Ahmed tube implantation in Asian eyes. *Journal of Glaucoma*.

[36] Law S. K., Nguyen A., Coleman A. L., Caprioli J. (2005). Comparison of safety and efficacy between silicone and polypropylene Ahmed glaucoma valves in refractory glaucoma. *Ophthalmology*.

